# Cefotaxime removal enhancement via bio-nanophotocatalyst α-Fe_2_O_3_ using photocatalytic degradation technique and its echo-biomedical applications

**DOI:** 10.1038/s41598-022-14922-3

**Published:** 2022-07-13

**Authors:** Mostafa F. Al-Hakkani, Gamal A. Gouda, Sedky H. A. Hassan, Mohammed S. Saddik, Mohamed A. El-Mokhtar, Maggie A. Ibrahim, Mahmoud M. A. Mohamed, Adham M. Nagiub

**Affiliations:** 1 Department of Chemistry, Faculty of Science, Al-Azhar University, Assiut Branch, Assiut, 71524 Egypt; 2 Department of Chemistry, Faculty of Science, New Valley University, El-Kharja, 72511 Egypt; 3 Department of Biology, College of Science, Sultan Qaboos University, 123 Muscat, Oman; 4 Department of Botany and Microbiology, Faculty of Science, New Valley University, El-Kharja, 72511 Egypt; 5 Department of Pharmaceutics and Clinical Pharmacy, Faculty of Pharmacy, Sohag University, Sohag, 82524 Egypt; 6 Department of Medical Microbiology and Immunology, Faculty of Medicine, Assiut University, Assiut, 71515 Egypt

**Keywords:** Biocatalysis, Chemistry, Materials science, Nanoscience and technology

## Abstract

The present paper evaluates the photocatalytic degradation (*PCD*) performance of the biofabricated hematite nanoparticles (*α-HNPs*) for the degradation approach of the Cefotaxime (*Cfm*). The optimum pH of the solution to achieve the best *PCD* was found to be 10.5. The kinetics study for the *PCD* of the *Cfm *via* α-HNPs* has been investigated and the reaction was found to be fellow pseudo-first-order at R^2^ = 0.992. The mass loading impact of *α-HNPs* was investigated and estimated for the maximum degradation of *Cfm* 0.4 mg/mL. UV–Vis confirmed that *α-HNPs* had a direct transition bandgap at 3.78 eV at a maximum absorption wavelength of 362 nm with suspension stability for 7 days. The probable mechanism of the *Cfm PCD *via* α-HNPs* and the degradation pathway was conducted. The validation of the suspension stability of the *α-HNPs* (−68.6 ± 11.8 mV) was determined using the zeta potential investigation test. XRD investigation was conducted after *Cfm PCD* showing an average crystallite size of 27.0 nm. XRD, TEM, SEM, EDX, and FT-IR analyses have been conducted for the *α-HNPs* before and after *Cfm PCD* confirming the high efficiency for the reusability of the current biocatalyst *α-HNPs* for further use. TEM results of the particle sizes of *α-HNPs* were found at 19.2 ± 4.4 and 20.6 ± 7.4 nm respectively before and after *Cfm PCD*. The efficiency of the *Cfm PCD* was found to be 99.1% after 6 h. High potent as an antibacterial agent of *α-HNPs* was investigated either *α-HNPs* alone or after its *PCD* activity against *Cfm*. The antibacterial activity revealed high sensitivity, especially toward Gram-positive species indicating its promising ability against pathogenic issues. Interestingly, *Cfm@α-HNPs* showed superior anti-proliferative activity as tested by MTT assay and were able to induce apoptosis in MCF7 and HepG2 cell lines using the flow cytometry technique at 20.7% and 17% respectively. Also, The IC_50_ of hydrogen peroxide scavenging was estimated and it was manifested that 635.8 and 665.6 μg/mL of *α-HNPs* before and after the *PCD* process of *Cfm* respectively.

## Introduction

The necessity to treat wastewater is a critical issue in the world especially. There are several ways for water to become polluted. Contamination from dyes, pharmaceuticals, and industrial waste is one of these sources^1^. Water containing organic pollutants such as industrial pharmaceutical waste and chemicals generated by textile industries has an impact on the biological cycle, specifically the photosynthesis process in plants, which has a direct impact on marine species and an indirect impact on human life^2^. Furthermore, polluted water has a significant influence on the life of birds, animals, and humans who are impacted by contaminated flowing water^3^.

**Figure 1:**
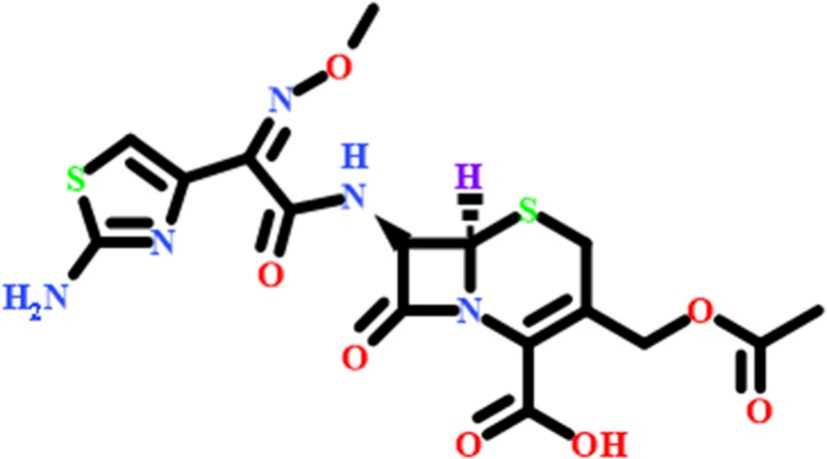
Structure of Cefotaxime (C_16_H_17_N_5_O_7_S_2_); molar mass: 455.46 g/mole.

Pharmaceuticals have been broadly spectrum used in the last four decades in human, sustainable agriculture, aquaculture, and vet application areas for bacterial infection treatment especially for enhancing the quality of human life^4^. Pharmaceuticals are an emerging environmental hazard due to the extremely large use of antibiotics that can easily be found in the aqueous environment, especially those that are classified as water-soluble drug substances. These drug constituents have been observed in drinking water^1,5,6^, surface water^7–9^, groundwater^9,10^, and sewage effluent^11–13^. The pharmaceutical drug substances can reach the aquatic environment via diversified sources such as pharmaceutical industrial wastewater^1,14^, Hospital drains^15^, and plants for conventional wastewater treatment^16,17^. Of the diverse pharmaceutical drug products, antibiotics have been of considerable environmental concern due to the emergence of antibiotic-resistant bacteria which couldn't be treated with currently recognized prescription, and the resulting rise in their chemical toxicity^1,18–20^. Cefotaxime (*Cfm*) is a typical example of an antibiotic. It is classified from the cephalosporin family of beta-lactam as one of their third-generation member Fig. 1. It has an antibiotic broad-spectrum activity as an antibacterial agent against either gram-positive or gram-negative micro-organism species^21^.

Many studies reported that the *Cfm* in the effluents after the wastewater treatment of the plants, hospitals drain, and sewage treatment facilities could be detected at a concentration greater than 300 ng/L^22–25^.

Although the toxicity of *Cfm* concentration in the aquatic environment is relatively low, the augmentation of resistant bacteria and resistance genes as a result of *Cfm* use is a more immediate issue^21^. Furthermore, because of its biodegradability, *Cfm* cannot be successfully removed by usual wastewater treatment techniques. As a result, innovative alternative solutions to the antibiotic contamination problem are necessary. León et al.^26^ reported a removal efficiency of 84.2% of 20 mg/L of *Cfm* removal photocatalytic method via 2.5 g/L of TiO_2_. Also, in a recent study mesoporous C_3_N_4_ was used as a photocatalyst by Mengmeng et al.^22^ in 1 g/L to remove the *Cfm* residual in a concentration of 2 mg/L in 60 min with an efficiency of 99%.

Several treatment approaches have been introduced to overcome this environmental issue, such as conventional methods such as biosorption, coagulation, membrane process, sedimentation, flocculation, filtration, and adsorption^1,27–29^. Owing to the unsatisfactory efficacy and often lack of utility of these methods, modern alternative advanced oxidation processes have arisen^30^. The later methods included different processes of chemical oxidation (O_3_, H_2_O_2_), photochemical (UV/H_2_O_2_), and photocatalysis (TiO_2_/ZnO/UV) that were used to generate the radical oxygen species (ROS) as hydroxyl radical (^•^OH). ROS has high activity as an oxidizing agent ageist divers of pharmaceutical compounds that have been successfully applied^31^. However, in commercial applications, the separation and recycling of ultrafine catalysts from treated wastewater are problematic, which could also pollute the treated water and is time-consuming and costly. These issues can be overcome by immobilizing the solid support *PCD*, which has an outstanding capacity to isolate Nano-sized materials and is cost-effective^32^. There is considerable interest in the wastewater treatment approach development that inevitably involves photocatalysts based on semiconductor nanomaterials especially transition metal oxides like BiVO_4_, WO_3_, ZrO_2_, TiO_2_, MnO_2_, Fe_2_O_3_, CuO, Cu/TiO_2_, ZnO, ZnO/Sb_2_MoO_6_, and C@Cu_2_O@Cu nanocomposite^1,3,33–39^.

Among all of these, Alpha Hematite Nanoparticles Fe_2_O_3_ (*α-HNPs*) offers several advantages such as low-cost, chemical stability, nontoxicity, and wide band gap^2,34^. To the best of our knowledge, we conduct herein for the first time photocatalytic of *Cfm* using *α-HNPs*. In this work, nanostructured *α-HNPs* (28.01 g/m^2^) were prepared as previously reported using a greener approach^36^. The resulting *α-HNPs* were characterized using various characterization techniques. The objectives of this investigation are to gain a mechanistic pathway in *PCD* of the *Cfm* wastewater-contaminant as an antibiotic model.

## Materials and methods

### Chemicals and reagents

All chemicals used in the current work were analytical grade. Methanol HPLC grade from (Merck). *Cfm* standard (C_16_H_17_N_5_O_7_S_2_); molar mass (455.46 g/mole); purity (96.3%) Zhuhai United Laboratories Co. Ltd (India) was kindly supplied by UP pharma (Assiut, Egypt). Disodium hydrogen phosphate anhydrous, Hydrochloric acid, Phosphoric acid 85%, Sodium hydroxide, and Hydrogen peroxide (Scharlau, Spain). Deionized water used in the analysis was prepared by reverse osmosis and passed through a 0.45 μm Millipore filter (Millipore Company, USA) before use.

### Instrumentation

UV–vis absorption measurement of the *α-HNPs* absorption was recorded in the range 200–800 nm using PerkinElmer [LAMBDA 40] Spectrophotometer using a quartz cell of 1 cm path length at room temperature. Malvern Zeta Sizer equipped with a 4mW helium/neon laser wavelength equals 633 nm at 25 °C (Nano-ZS). The XRD parameters as crystallite size “D” could be determined using the Scherrer equation for each peak. Also, the other XRD parameters could be estimated as the strain of the lattice (ℇ), placing distance (d), dislocation density (δ) and stacking fault (α), crystallinity (%), and crystallinity index (CI) as manifested in the supplementary material file; Eqs. (1–7)^3,36,37^. FT-IR analysis was recorded on a Thermo Fisher [Nicolet iS10 FT-IR spectrometer] in a wavenumber range of 4000–400 cm^−1^ using an ATR module. The particle size of the *α-HNPs* before and after *Cfm PCD* was conducted using transmission electron microscopy [TEM; JEOL JEM-100C XII)]. The morphology and chemical composition of the *α-HNPs* and *Cfm@α-HNPs* were investigated using scanning electron microscopy [SEM; JSM IT 200]. *Cfm* quantitative analysis and its related substances were determined using the LC-20A HPLC instrument with the PDA (Shimadzu). The method was performed on the (Thermo Scientific) RP column BDS (150 mm × 4.0 mm × 5 μm) with a PDA detector at 235 nm, column oven at 30 °C, and injection volume of 20 μL. Flow cytometry was carried out using FACS-Calibur flow cytometer (Becton Dickinson, USA) and data were analyzed using FlowJo software (Treestar, Ashland, OR).

### Methods and experiments

The photocatalytic efficiency studies were conducted under direct sunlight irradiation from 11.00 AM to 5.00 PM. All the study experiments were implemented according to the previously reported *Cfm* HPLC analysis method by Al-Hakkani^21^. As a general procedure, after any process of *Cfm* solution and *α-HNPs* treatments the samples were filtered using nylon filter paper with a pore size of 0.45 µm using the Buckner filtration system. Then as an assurance procedure, an additional filtration step was performed using a syringe filter of 0.2 µm before introduction to the HPLC for analysis. To identify the related substances that were produced from the *Cfm* degradation; individual impurities (A, B, C, E, and F) were injected under the same analysis circumstances according to European Pharmacopoeia^21,40^. The resolution between the nearest adjacent peak and the *Cfm* principle peak should be more than 1.5 according to the guidelines of the analytical method validation protocols^21,33,41–44^.

#### Batch mode experiments

The placebo samples “*Cfm* only without any processes applied as adsorption, photolysis or *PCD*s” were conducted as a zero time for each experiment individually for each parameter for the whole of the study. Also, samples after 2 h of adsorption in the dark under continuous stirring at 350 rpm were conducted before starting the photocatalytic study to exclude the adsorption/desorption effect.

The degradation % was calculated using the equation in the supplementary material file; Eq. (8).

#### Interactive effect of solution pH change

Lopez-Alvarez et al.^45^ reported that the most important parameters affecting the *PCD* process are the pH of the solution and catalyst dose. So, both of them and the time effect parameter were to be evaluated.

The photolysis and photocatalytic studies were conducted for 6 h via direct sunlight for 250 mL of *Cfm* 20 mg/L using 250 mg of as-biofabricated *α-HNPs* with continuous stirring at 350 rpm. The impact of solution pH on the *Cfm* degradation was implemented firstly by maintaining all the parameters fixed at different pH medium solutions in the range (2.5–12.5). Hydrochloric acid 0.1 M and sodium hydroxide 0.1 M were used for the pH adjustment. The pH of the solution was adjusted before starting the sunlight irradiation and is not controlled while conducting the degradation reaction.

#### Kinetic mechanism studies

To determine the time effect “kinetic study profile”, 250 mL of *Cfm* 20 mg/L at pH 10.5 samples were prepared in a beaker containing 250 mg of the as-biofabricated *α-HNPs* under continuous stirring at 350 rpm. At different time intervals (1, 2, 3, 4, 5, and 6 h); the degradation % of *Cfm* was estimated.

The rate constants of the *Cfm PCD* can be estimated via the pseudo-first-order and second-order equations as in the supplementary material file; Eqs. (9,10).

#### Influence of the as-biofabricated *α-HNPs* catalyst loading

For the investigation study of the as-biofabricated *α-HNPs* mass on the photolysis and photocatalytic activities of *Cfm*, 250 ml of *Cfm* solution 20 mg/L at pH 10.5 samples were prepared. Solutions were taken individually in a beaker at different quantities of *α-HNPs* at (0.04–1.0 g/L). Then the samples were exposed to direct sunlight with continuous stirring at 350 rpm.

### Practical application using an actual pharmaceutical wastewater sample after production of *Cfm*

Initially and before the photocatalytic process application, the physicochemical parameters as solution pH, conductivity, total dissolved solids (TDS), and zero-time HPLC assay were analyzed. In a 100 mL beaker; 50 mL of an actual pharmaceutical wastewater sample that was previously filtered through a nylon membrane filter of 0.45 µm, the *α-HNPs* were added to get a concentration of 1.0 mg/mL with continuous stirring for 2 h at 350 rpm at room temperature. Consequently, the sample was left in direct sunlight from 11:00 AM to 04:00 PM. After completion of the reaction, the sample was filtered using nylon filter paper with a pore size of 0.45 µm using the Buckner filtration system, then an additional filtration step was performed using a syringe filter of 0.2 µm before introduction to the HPLC instrument for analysis.

### Antibacterial activity

Four examples were used for the investigation of the antibacterial activity of the as-prepared *α-HNPs* against *Cfm@α-HNPs* after *Cfm* degradation. Gram-negative bacteria species were tested as *Escherichia coli* (*E. coli*) and *Salmonella typhimurium* (*S. typhimurium*). While *Enterococcus faecalis* (*E. faecalis*) and *Staphylococcus albus* (*S. albus*) were testes as examples of Gram-positive bacteria. The bacterial types were kindly provided by the lab of Microbiology; Faculty of Science; New Valley University, Al-kharga, Egypt. The antibacterial activity was investigated via the agar well diffusion according to Magaldi et al.^1,46^. In brief, the wells were made by punching the nutrient agar surface with the sterile cork borer (8 mm diameter). The Dimethyl sulfoxide (DMSO) was used as a negative control where all testes were suspended in DMSO as a solvent. Cefotaxime sodium (Cephalosporin third-generation antibiotic) 250 µg/mL was used as a positive control. *Echinacea purpurea* (*E. purpurea*) and the as-prepared *α-HNPs* against *Cfm@α-HNPs* after *Cfm* degradation were tested at a concentration of 1000 µg/mL. All tests were added to the wells. The Petri dishes were incubated at 30° C for 24 h, and the clear inhibition zones were estimated in mm and recorded.

### Anti-proliferative activity

MTT assay was used to evaluate the effect of the different compounds on the proliferation of MCF7 cells (breast carcinoma cell line) and HepG2 cells (Human liver Hepatoma carcinoma cell line). About 5 × 10^4^ cells were inoculated in wells of 96 well tissue culture plates. Then cells were treated with serial dilutions of the tested samples and incubated at 37° C overnight. MTT solution (Promega, USA) was applied at the recommended concentration to cells for 3 h and the formed formazan crystals were solubilized. The optical density was measured at 560 nm and the anti-proliferative activity of *E. purpurea* liquid extract, *α-HNPs*, and *Cfm@α-HNPs* were evaluated against control untreated cells as previously described.

To confirm these results, MCF-7 cells were treated with the *E. purpurea* liquid extract, *α-HNPs* and *Cfm@α-HNPs* at a concentration of 100 µg/ml for 24 h, and apoptotic cells were evaluated using flow cytometry. Following incubation with the indicated test samples, cells were stained with Annexin V-FITC and propidium iodide (FITC Annexin V Apoptosis Detection Kit with PI, Biolegend, USA) to evaluate the frequency of late apoptotic cells which acquired both dyes.

### Hydrogen peroxide scavenging (H_2_O_2_) assay

The ability of as-biofabricated *α-HNPs* to scavenge hydrogen peroxide can be determined according to the reported method by Ruch et al.^47^. A solution of 0.004 M of hydrogen peroxide was prepared using the 0.05 M phosphate buffer pH 7.4 solution as a solvent. The blank solution was conducted using a phosphate buffer solution in absence of the hydrogen peroxide. The standard hydrogen peroxide concentration was estimated spectrophotometrically via absorption measurement at 230 nm as (A_S_). The as-biofabricated *α-HNPs* were suspended in the distilled water subsequently, they were added to the hydrogen peroxide/phosphate buffer in a final concentration range (10–1000 μg/mL). Finally, the absorbance at 230 nm was determined after 10 min as (A_T_).

The scavenging percentage of hydrogen peroxide could be calculated as in the supplementary material file; Eq. (11).

## Results and discussion

The retention time (R_t_) of the *Cfm* peak was found to be about 12.5 min. Also, the Rt of *Cfm*-related substances was determined and found to be at about 2.9, 4.4, 8.2, 9.6, and 14.6 min for impurities B, C, E, A, and F respectively.

**Figure 2:**
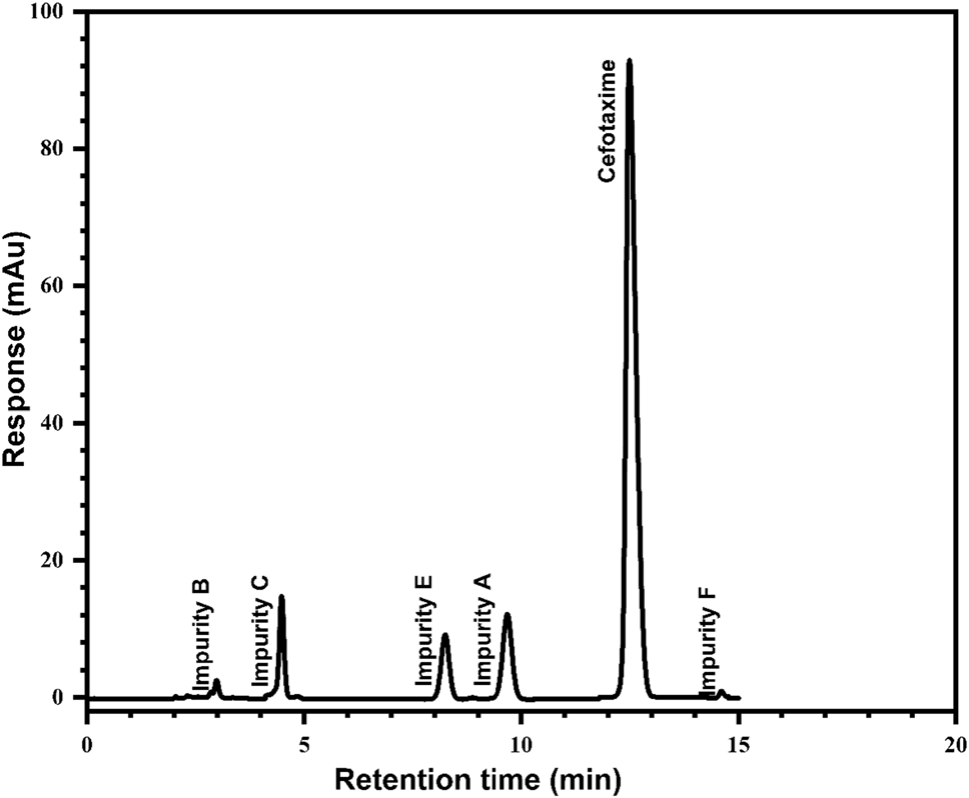
Cefotaxime and its related substances HPLC chromatogram.

The *Cfm* principle peak was found to be resoluted from the impurity F at an excellent value via 8.11 as revealed in Fig. 2. Any other impurity less than the reporting level at 0.05% of the *Cfm* peak area in the chromatogram will be disregarded according to the European Pharmacopoeia guideline^40^.

### Interactive effect of solution pH change

The pH of the solution in diverse photocatalytic systems is a factor that can affect the polarization of the catalyst and the dissociation of the substrate, which can determine the various electrostatic interactions among the present species in the reaction medium^1,48,49^.

To study the solution pH effect on the degradation of *Cfm*; some experiments were implemented in solution pH within the range of 2.5–12.5 maintaining the rest of the experiment factors fixed at 1.0 g/L of *α-HNPs* loading dose and 250 mL of 20 mg/L of *Cfm* concentration for 6 h.

**Figure 3:**
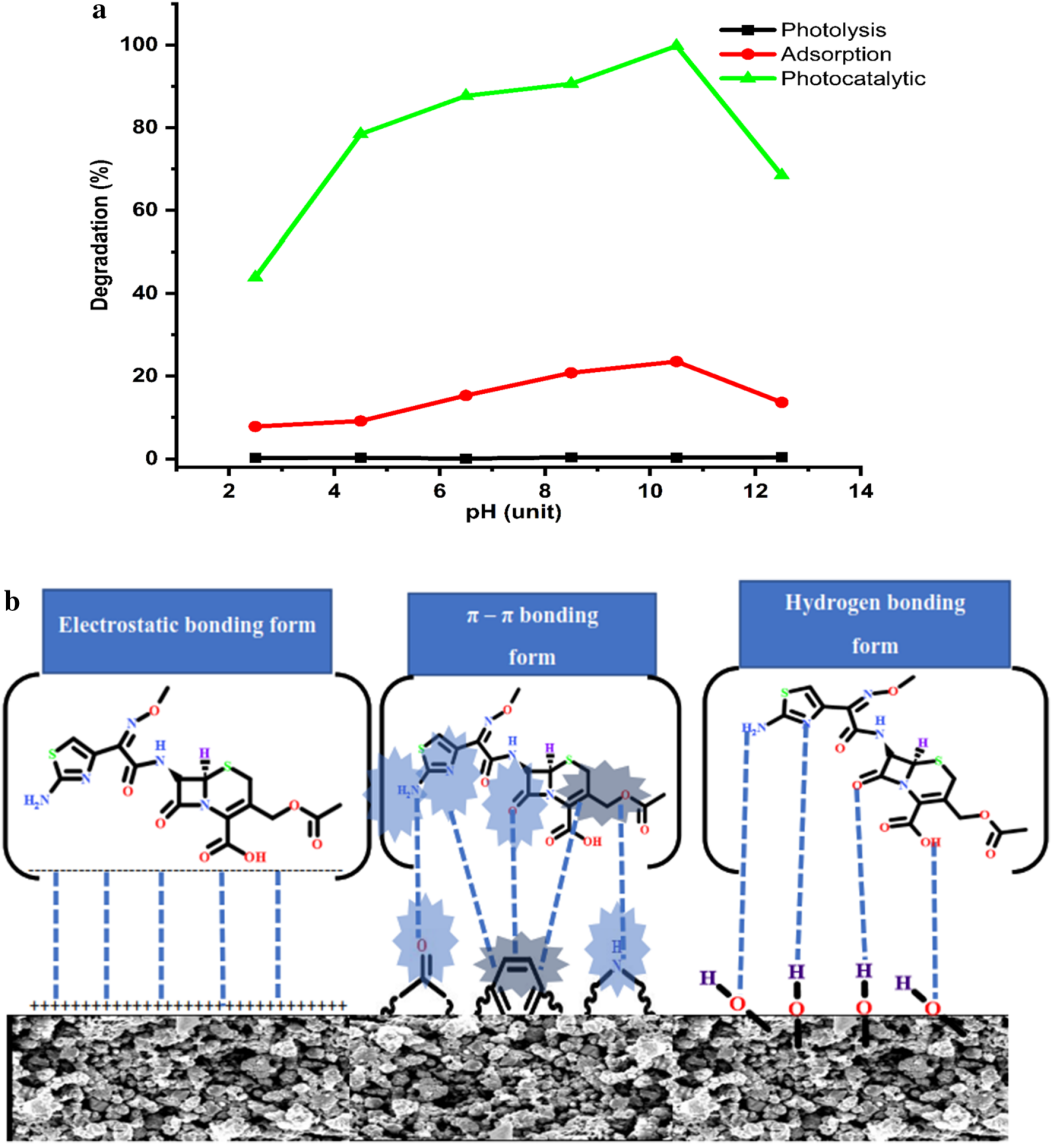
(**a**) Cefotaxime degradation (%) against pH solution change effect. (**b**) Cefotaxime/*α-HNPs* binding form probabilities.

According to Fig. 3a; the comparison profiles for both photolysis and photocatalytic for *Cfm* degradation; it can be concluded that there is no effect of the sunlight alone on the *Cfm* degradation over at the full pH range 2.5–12.5. On the other hand, the photocatalytic of the *Cfm* degradation increased dramatically (43.9–90.6%) in the pH range 2.5–8.5 showing the maximum degradation of *Cfm* (99.8%) at pH 10.5 followed by decreasing at pH 12.5 (68.5%).

The *Cfm* degradation usually depends on the adsorption of its molecules onto the *α-HNPs* surface and the generation of the hydroxyl free-radical which depends on the availability of hydroxyl ions in the reaction medium. As a result of the previous determination of the zero-point charge (pH_zpc_) of *α-HNPs* that was found to be 5.2^36^. So, at the pH of solutions below this value; the *α-HNPs* surface will be positively charged. On the contrary, at pH solutions more than pH 5.2, the *α-HNPs* surface will be negatively charged. *Cfm* shows three pK_a_ (acid dissociation constant) values at 2.1, 3.4, and 10.9 as León et al. reported^50^. So, the *Cfm* at pH range (4–10.5) presents as negatively charged ions.

According to the attraction electrostatic force among the *α-HNPs* surface charges and the *Cfm* ions under the dedicated pH reaction medium (2.5–4.5), the promotion to generate the hydroxyl radicals was enhanced. The increase in *Cfm* degradation efficiency % at the pH range (6.5–10.5) may be attributed to hydrogen bonding formation between the *Cfm* and the adsorbed bioactive molecules that surrounded the as-biofabricated *α-HNPs* or π-π interaction^1,36,37^ as manifested in Fig. 3b.

Completely *Cfm* dissociation at pH 12.5 or more causes the presence of the *Cfm* species as deprotonated anions and the negatively charged surface of the as-biofabricated *α-HNPs* maybe it is the main cause of the repulsion electrostatic force. That was subsequently, followed by a decrease in *Cfm* degradation.

### Kinetic mechanism studies

**Figure 4:**
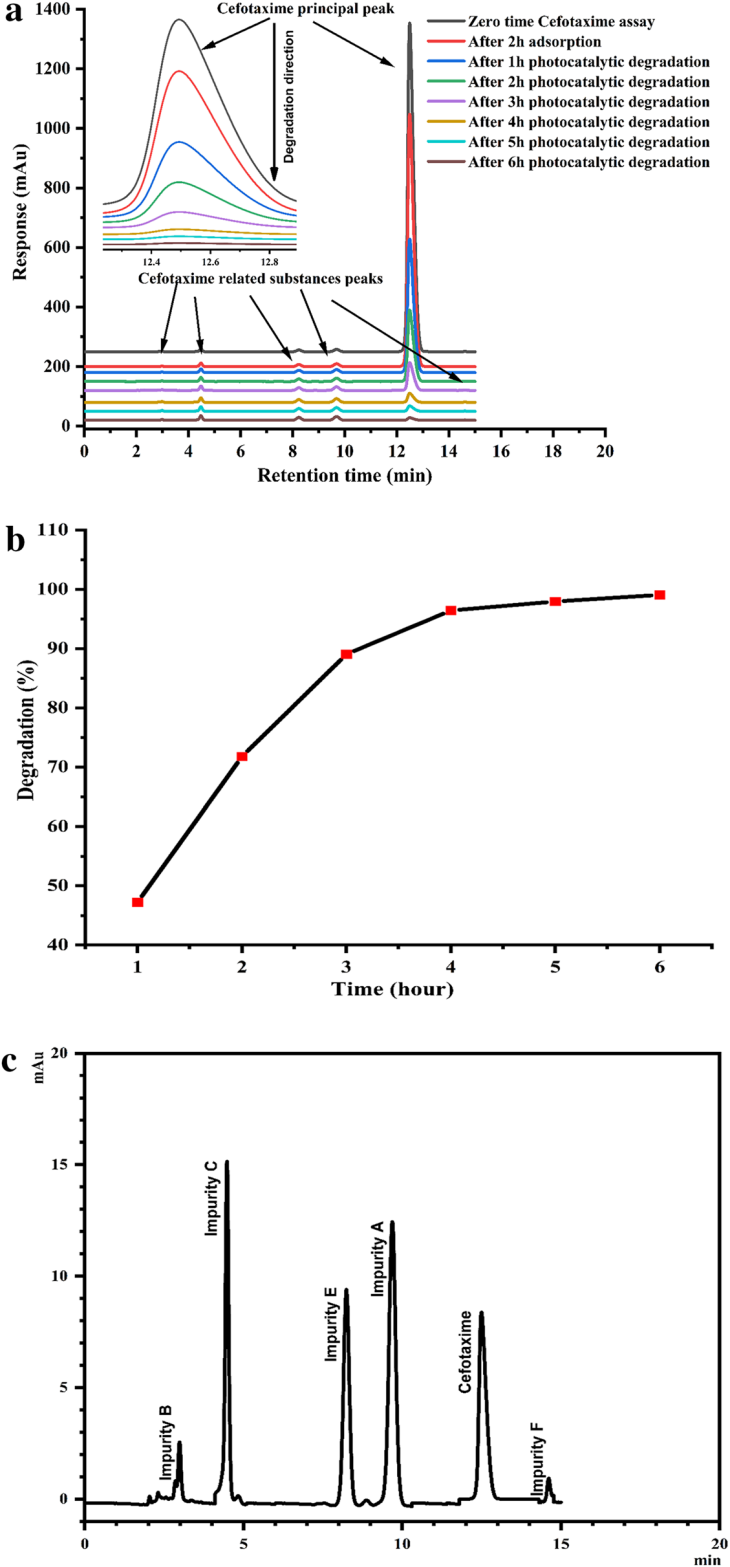

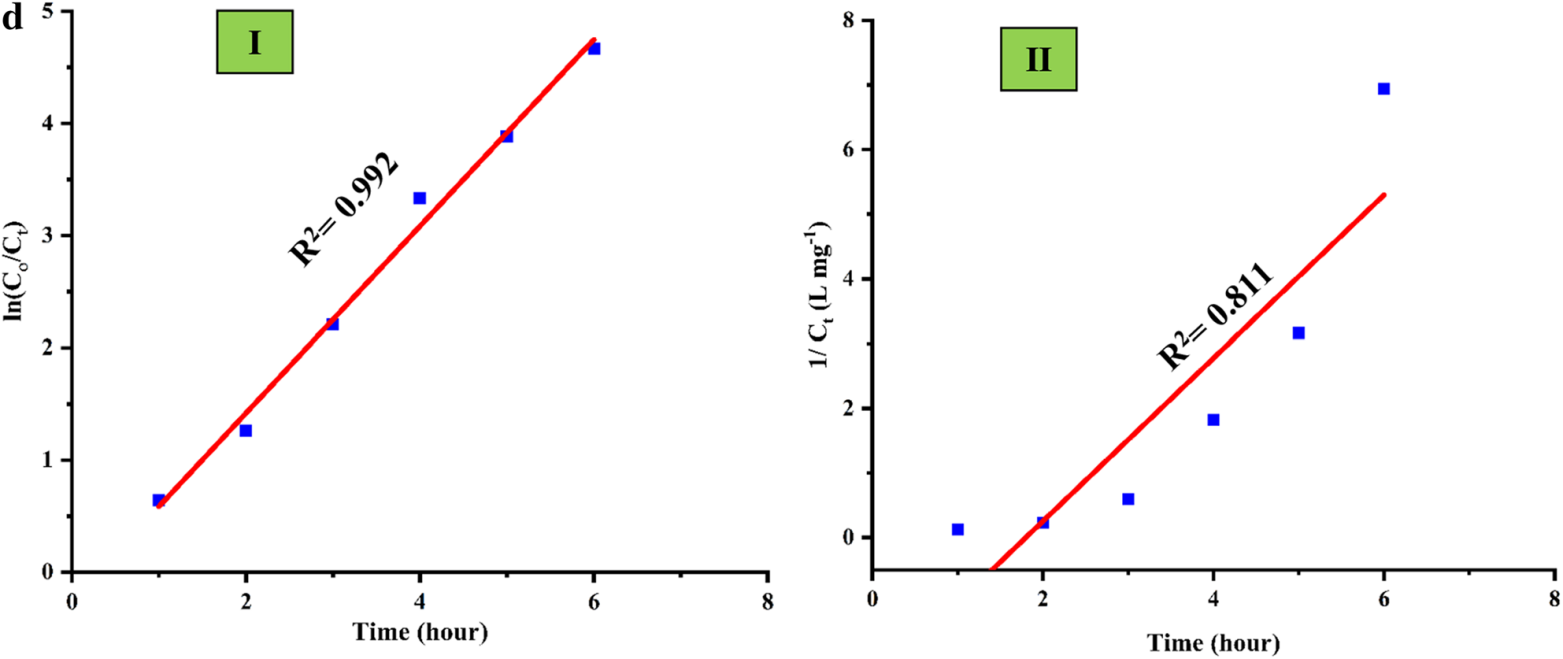
(**a**) Cefotaxime degradation (%) against different time intervals using *α-HNPs*. (**b**) Cefotaxime degradation (%) profile as a function in time. (**c**) Cefotaxime photocatalytic degradation after six hours via* α-HNPs* (HPLC-related substances profile). (**d**) Kinetic models I) Pseudo-first-order, II) Second-order.

The *Cfm* responses at zero time, after adsorption, and after the *PCD* at different time intervals (1–6 h) were manifested in Fig. 4a. It is very clear that the efficacy of the as-biofabricated *α-HNPs* on *Cfm* degradation as a photocatalytic agent. The *Cfm* peak response was decreased rapidly as a function of degradation time.

The first rapid *PCD* was observed at the first hour of the reaction and it was found to be 47.2%. The increase of *PCD* efficiency almost reached the maximum after 4 h at (96.4%). After that, the progress in the degradation was limited where it was increased up to 97.9% followed by a slight increment at 99.1% after further 2 h of sunlight time exposure as depicted in Fig. 4b. So, the critical photocatalytic time interval is represented after 4 h of the reaction.

As the HPLC degradation profile manifested (Fig. 4c and Table 1); the *Cfm* further degradation resulted from related substances (impurities A, B, C, E, and F) showed a notable increase in their responses. The most abounded of these related degradant substances was impurity C which gave an increased assay response factor at a ratio from < 0.05% at *Cfm* zero-time assay to 1.36% at the end of catalytic degradation after 6 h. Impurity A came in the second-order of the increase in its assay response by 1.32% from the *Cfm* zero-time assay. Impurities E, B, and F came posteriorly in degradation ratios assay as 1.00%, 0.27%, and 0.14% respectively.

**Table 1:** HPLC *Cfm* related substances impurities profile data after six hours of *PCD *via* α-HNPs.*

Impurity	IUPAC Name	Chemical name	Molecular formula	Molar mass (g/mol)	R_t_ (min)	Degradation (%)
A	(6R,7R)-7-((Z)-2-(2-aminothiazol-4-yl)-2-((methoxy-d3)imino)acetamido)-3-(methoxymethyl)-8-oxo-5-thia-1-azabicyclo[4.2.0]oct-2-ene-2-carboxylicacid	Cefetamet	C_14_H_15_N_5_O_5_S_2_	397.43	9.601	1.32
B	(6*R*,7*R*)-7-[[(2Z)-2-(2-aminothiazol-4-yl)-2-(methoxyimino)acetyl]amino]-3-(hydroxymethyl)-8-oxo-5-thia-1-azabicyclo [4.2.0]oct-2-ene-2-carboxylic acid	Deacetyl Cefotaxime	C_14_H_15_N_5_O_6_S_2_	413.43	2.934	0.27
C	(6*R*,7*R*)-3-[(acetyloxy)methyl]-7-[[(2*Z*)-2-[2-(formylamino)thiazol-4-yl]-2-(methoxyimino)acetyl]amino]-8-oxo-5-thia-1-azabicyclo [4.2.0]oct-2-ene-2-carboxylic acid	N-Formylcefotaxime	C_17_H_17_N_5_O_8_S_2_	483.48	4.458	1.36
E	(5a*R*,6*R*)-6-[[(2*Z*)-2-(2-aminothiazol-4-yl)-2-(methoxyimino)acetyl]amino]-5a,6-dihydro-3*H*,7*H*-azeto[2,1-*b*]furo[3,4-*d*][1,3]thiazine-1,7(4*H*)-dione	Deacetylcefota-xime Lactone	C_14_H_13_N_5_O_5_S_2_	395.41	8.272	1.00
F	(6*R*,7*R*)-3-[(acetyloxy)methyl]-7-[[(2*Z*)-2-[2-[[[(6*R*,7*R*)-7-[[(2*Z*)-2-(2-aminothiazol-4-yl)-2-(methoxyimino)acetyl]amino]-2-carboxy-8-oxo-5-thia-1-azabicyclo[4.2.0]oct-2-en-2-yl]methyl]amino]thiazol-4-yl]-2-(methoxyimino)acetyl]amino]-8-oxo-5-thia-1-azabicyclo[4.2.0]oct-2-ene-2-carboxylic acid (	Cefotaxime Dimer	C_30_H_30_N_10_O_12_S_4_	850.88	14.649	0.14

It is clear that the total impurities assay was about 3.82% of the principle *Cfm* peak, this ratio reflects the strength of the as-biofabricated *α-HNPs* toward *Cfm* degradation for its primary components as carbon dioxide and water.

Figure 4d shows that the *Cfm* degradation fits appropriately with first-order kinetic according to the R^2^ value which was found to be 0.992. The rate constant of the *Cfm* was found to be 0.8325 h^−1^.

### Influence of the as-biofabricated *α-HNPs* catalyst loading

It is clear to man that without using the *α-HNPs* catalyst (photolysis) there was no significant degradation change over the reaction time of 6 h and it can be neglected as shown in solution pH and time change effect.

**Figure 5:**
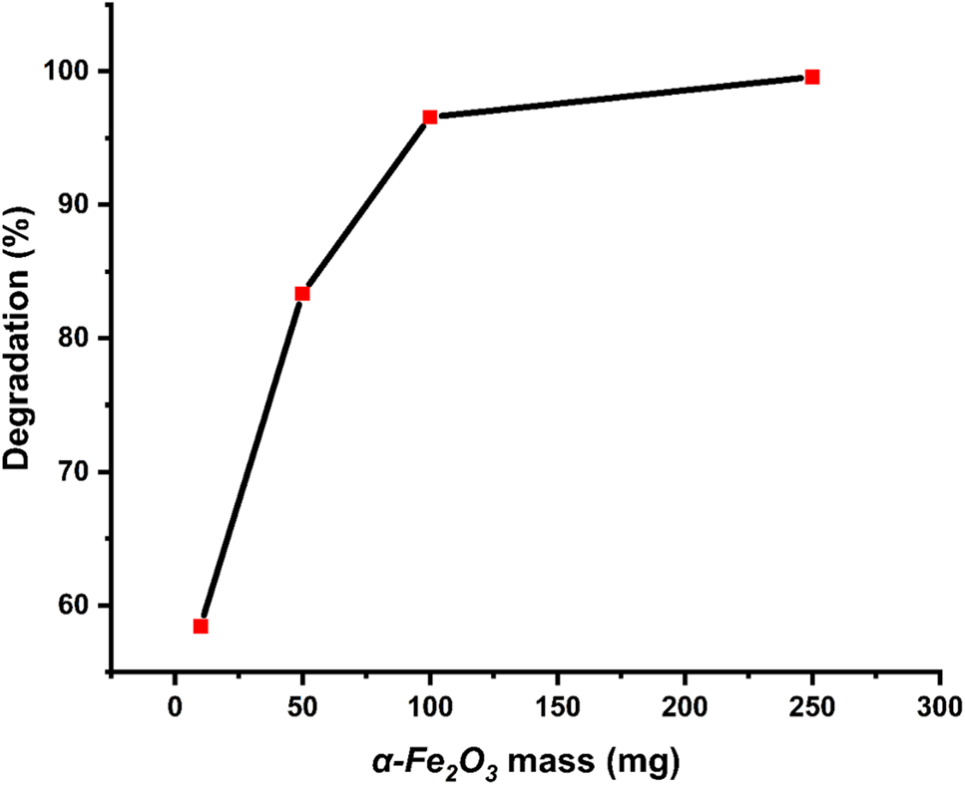
Cefotaxime degradation (%) against adsorbent of *α-HNPs* mass effect.

For studying the effect of the as-biofabricated *α-HNPs* loading, a series of experiments were conducted for 4 h at different doses of the *α-HNPs* in the range 0.04 to 1.0 g/L maintaining the *Cfm* concentration at 20 mg/mL and pH of 10.5 as shown in Fig. 5.

The photocatalytic is a surface phenomenon so, it strongly depends on the crystallinity nature and the surface area “28.01 g/m^2^ as previously reported in our approach^36^” that is proportional directly to the mass loading of the catalyst under study. According to the increase of the as-biofabricated *α-HNPs* loading, along with reaction time, there was a drastic rise in the degradation rate (58.5%, 83.4%, 96.6%, and 99.6%). So, it was found to be the degradation profile is properly dose-based. The increment efficiency of degradation may be attributed to the photon absorption via the as-prepared *α-HNPs* that cause the generation of the hole-electron pairs^1^. So, the total activated surface area increased with increasing the catalyst dose which subsequently, increase the reactive oxygen species production as revealed in the photocatalytic degradation mechanism scheme equations. Any increase in the *α-HNPs* after 0.4 g/L loadings had no notable effect on the increase of *Cfm* degradation efficiency compared with the loading dose effect at (0.04–0.2 g/L). So, it can be negligible because it was an almost slight increase. This behavior may be due to the agglomeration of NPs, solution opacity, or scattering of the light at higher doses of the as-prepared *α-HNPs*^51^. So, 0.4 g/L can be selected as a convenient dose for further studies.

### Photocatalytic activity comparison studies of the biosynthesized *α-HNPs*

From the last decade and up to date, the biofabricated *α-HNPs *via various parts of different kinds of plants were paid attention to and gained great interest in several applications, especially in photocatalytic activity against some of the organic substances as reported in Table 2.

**Table 2:** Photocatalytic activity of the biosynthesized *α-HNPs *via various parts of different kinds of plants.

Plant name/part	Substrate	References
*Amaranthus dubius* leaf	Methylene orange dye	^52^
*Cyperus rotundus* L	Congo red dye	^53^
*Curcuma* *Tea* leaves	Methyl orange dye	^54^
*Cynometra ramiflora* fruit	Methylene blue dye	^55^
*Pomegranate* (*Punica granatum*) seeds	Blue 4 dye	^56^
*Mandarin* peels *“Citrus reticulum*”	Basic Maxilon Blue GRL, acidic Neolan Blue 2G dyes, and 2,6-dichlorophenol	^57^
*Cynometra ramiflora*	Rhodamine-B dye	^58^
*Carica papaya leaf*	Remazol yellow RR dye	^59^
*E. purpurea*	Cefotaxime	Current study

### UV–Vis analysis

**Figure 6:**
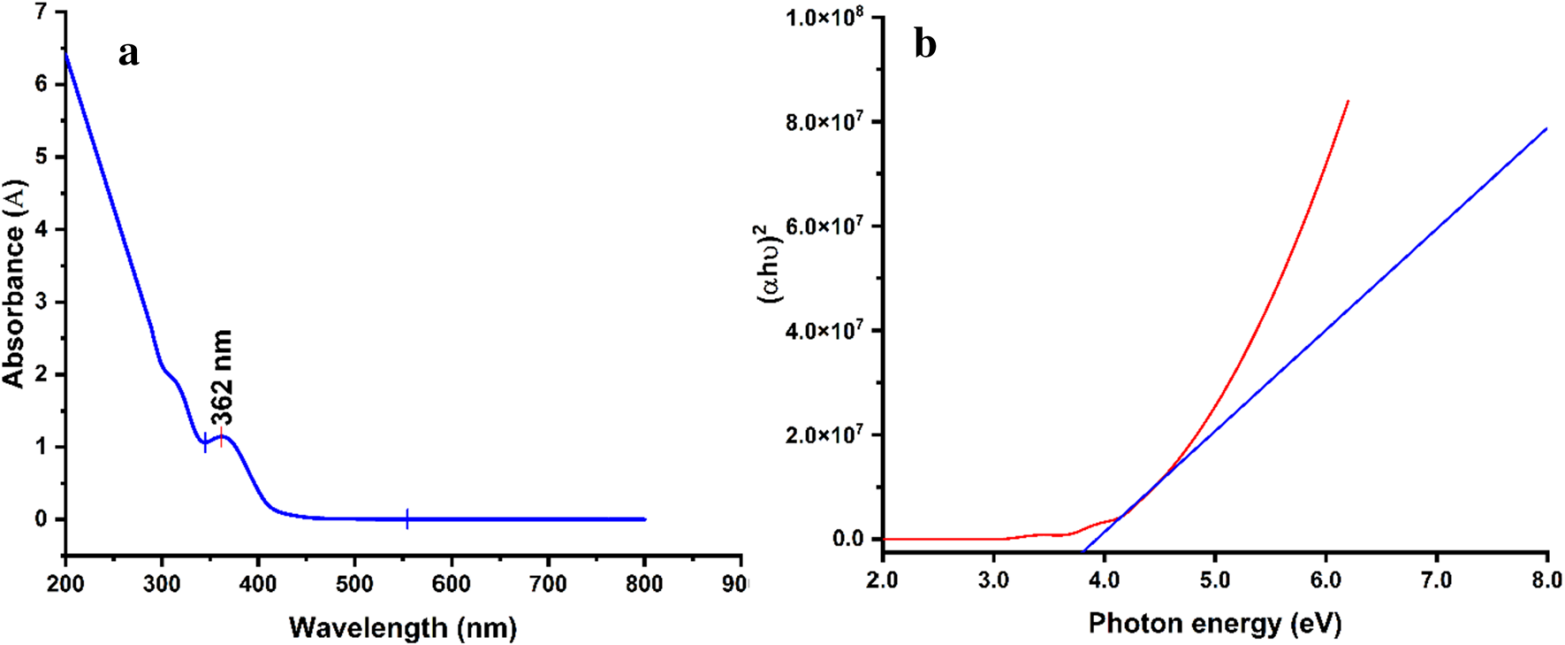
Spectra of the as-biofabricated *α-HNPs* (**a**) UV–Vis excitation absorption, (**b**) Tauc plot of direct transition energy bandgap.

The optical characterizations of the biosynthesized *α-HNPs* powder sample were investigated after it was dispersed in deionized water. The *α-HNPs* exhibited the maximum absorption peak in the UV range at 362 nm Fig. 6a, this result was found to be compatible with the previously reported surface plasmon (SPR) peaks by Rajiv et al.^60^ and Al-Hakkani et al.^36^. The stability of the *α-HNPs* suspension form in the deionized water was tested for one week by examining for the distinct SPR peak in the optical spectrum and it was found to be at the same wavelength. The bandgap energy of the as-prepared *α-HNPs* could be determined using the optical as manifested in the supplementary material file; Eqs. (12, 13) ^3^. The E_g_ of the direct transition was estimated by extrapolating the linear portion of the plot of (αhυ)^2^ against hυ Fig. 6b. E_g_ was found to be 3.78 eV which was agreed with the previous findings by Al-Hakkani et al.^36^ and Sharma et al.^61^.

### Zeta potential

**Figure 7:**
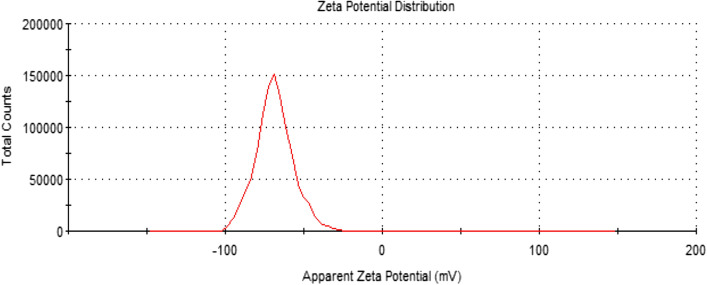
Zeta potential of the as-biofabricated *α-HNPs*.

The suspension stability of the *α-HNPs* could be estimated using the value of the zeta potential instrument^3,36^. The value of zeta potential of *α-HNPs* suspension stability in water was found to be −68.6 ± 11.8 mV which indicated that NPs had an excellent stability Fig. 7.

Zeta potential result was found to be compatible with the UV–Vis. *α-HNPs* suspension stability. Several studies reported that the colloidal/suspended particles had good stability if their charged surface passed the critical value ± 30 mV. Table 3 shows the relationship between the zeta potential value of the colloidal/suspended material and its corresponding relative stability. Highly positive or negative values onto the charged surface generate major repulsion forces, whilst repulsion between particles with the same electrical charge inhibits the particle agglomeration and hence it gives good dispersibility^1–3,36,62,63^.

**Table 3:** Zeta potential value stability indications.

Zeta potential value (-mV)	Colloidal/suspension stability indication	Zeta potential value (+ mV)
−10, … , 0	Flocculation or rapid coagulation	0, …, + 10
−30, … , −10	Relative (onset)	+ 10, …, + 30
−40, … , −30	Moderate	+ 30, …, + 40
−60, … , −40	Good	+ 40, …, + 60
< −60	Excellent	> + 60

### Degradation mechanism

**Figure 8:**
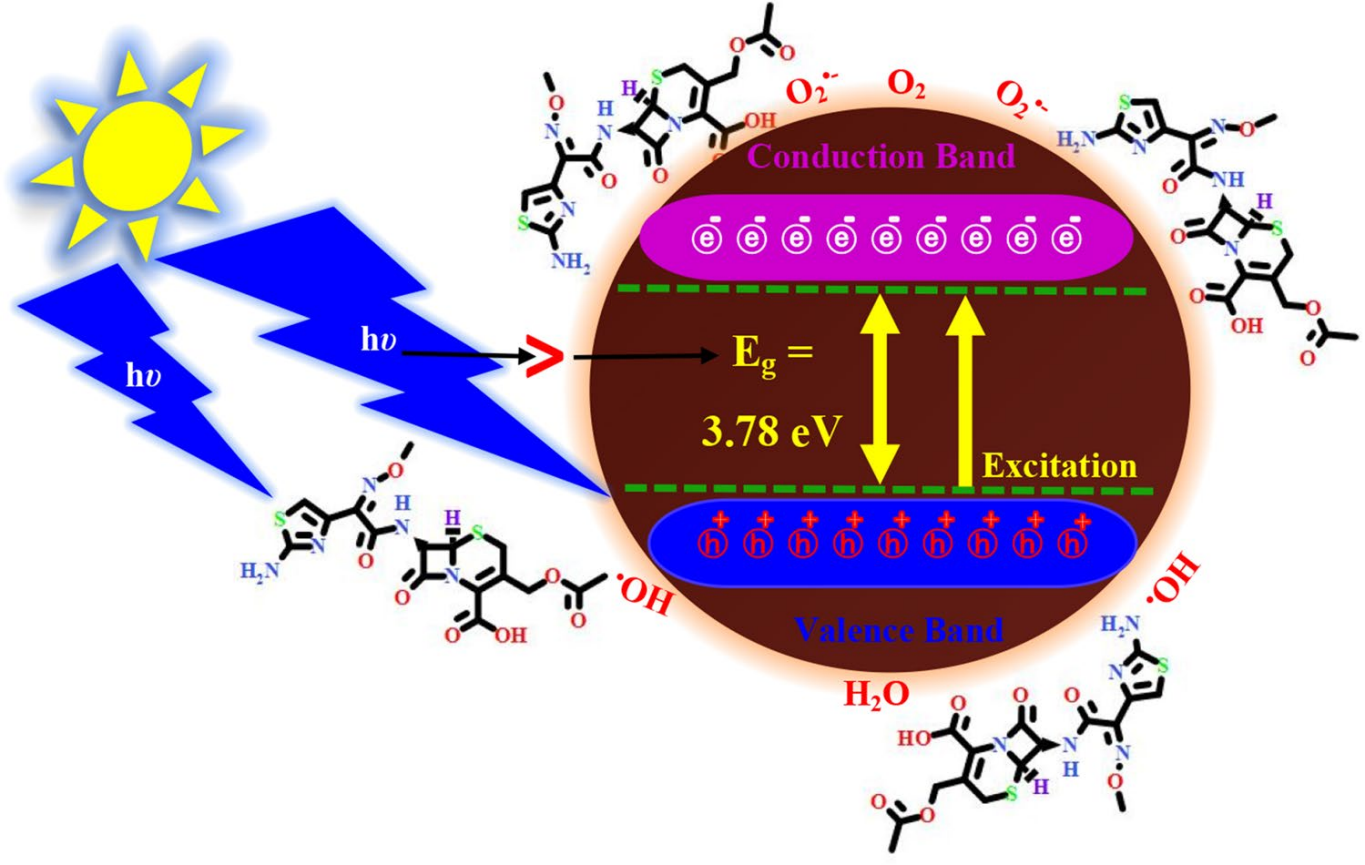
The Cefotaxime photocatalytic degradation probable mechanism via* α-HNPs.*

Sunlight irradiation-induced *α-HNPs* for production of the ROS especially hydroxyl radical and superoxide anion that is mainly responsible for *Cfm* degradation. The probable degradation mechanism can be illustrated in Fig. 8.

According to the smallest energy band gap of the as-biofabricated semiconductor *α-HNPs* (3.78 eV) and also as a previously reported^36^, it can be easily excited via direct sunlight in the visible range and this is the main cause of the NPs photocatalytic activity as summarized in photocatalytic degradation mechanism scheme Eqs. (1–15). Briefly, when most of the NPs were irradiated under the sunlight induction, the electrons were being promoted to the highest energy level leaving the valence band. The hole-electron pairs were being generated; holes occupy the valence band while electrons locate in the conduction band^33^.

Firstly, the holes (*h*^+^) cause hydrolysis of water molecules to protons and hydroxyl ions which are subsequently, converted to hydroxyl radical. The ROS as hydroxyl radical considers the motive force in the oxidation of organic compounds where it acts as an oxidizing agent under convenient conditions. The final photocatalytic products are the primary components of any organic substances; that may undergo further degradation resulting water, carbon dioxide, and inorganic anions.

On the other hand, the negative charges (*e*^*−*^) that are present in the conduction band induce the conversion of the adsorbed oxygen gas molecules to superoxide radicals which may directly degrade the organic substance or combined with the protons or holes forming an active hydroxyl radical species again and peroxides^57^. Finally, we can report that the various bioactive molecules present in the *Echinacea* extract which adsorbed at the NP's surface, and the NPs themselves act as catalysts to promote photocatalytic activity.

The formation/elimination probabilities of the ROS during the *PCD* mechanism of *Cfm *via* α-HNPs* can be explored as the following mechanism scheme Eqs. (1–15):1

2 $$ [h\upsilon > E_{g} ] + \alpha - HNPs \to \, e^{ - } + h^{ + } $$

3 $$ H_{2} O + h^{ + } \to H^{ + } + {}^{ - }OH\quad (Hydroxyl\;anion) $$

4 $$ {}^{ - }OH + h^{ + } \to {}^{\cdot }OH\quad { (}Hydroxyl\;radical{)} $$

5 $$ O_{2} + e^{ - } \to O_{2}^{\cdot - } \quad (Superoxide \, anion) $$

6 $$ O_{2}^{\cdot - } + e^{ - } \to O_{2}^{\cdot 2 - } \quad (Peroxide\;anion) $$

7 $$ O_{2}^{\cdot 2 - } + 2H^{ + } \to H_{2} O_{2} \quad (Hydrogen\;peroxide) $$

8 $$ O_{2}^{\cdot - } + H^{ + } \to {}^{\cdot }OOH\quad (Perhydroxyl \, radical) $$

9 $$ 2[{}^{\cdot }OOH] \to H_{2} O_{2} + O_{2} $$

10 $$ O_{2}^{\cdot - } + e^{ - } + H^{ + } \to {}^{\cdot }OH $$

11 $$ O_{2}^{\cdot - } + H_{2} O_{2} \to {}^{\cdot }OH + {}^{ - }OH + O_{2} $$

12 $$ H_{2} O_{2} + e^{ - } \to {}^{\cdot }OH + {}^{ - }OH $$

13 $$ H_{2} O_{2} + e^{ - } + H^{ + } \to {}^{\cdot }OH + H_{2} O $$

14 $$ {}^{ \cdot }OH + e^{ - } + H^{ + } \to H_{2} O $$

15

*Cfm PCD* mechanism can be summarized as shown in scheme Eq. (15); firstly, an interaction of the *Cfm* with the photon energy forms an intermediated activated substrate. Subsequently, the latter can interact with any of the ROS final species as O_2_/O_2_^•−^/^•^OH producing preoxylated or hydroxylated intermediate products that followed via degradation to the primarily related byproducts.

### Practical application using an actual pharmaceutical wastewater sample after production of *Cfm*

Water physicochemical characteristics and assay of the *Cfm* before and after photocatalytic process activity were evaluated and listed in Table 4.

**Table 4:** Water physicochemical characteristics and assay of the *Cfm.*

Characteristic parameter	Before	After
Concentration (mg/L)	9.7	Not detected
Conductivity (*μ*S/cm)	266.4	98.0
TDS (mg/L)	131.0	44.2
pH	8.16	6.81

The operating procedures revealed the efficiency of the photocatalytic activity in the industrial pharmaceutical wastewater treatment using *α-HNPs* as a promising photocatalytic agent.

### XRD analysis

**Figure 9:**
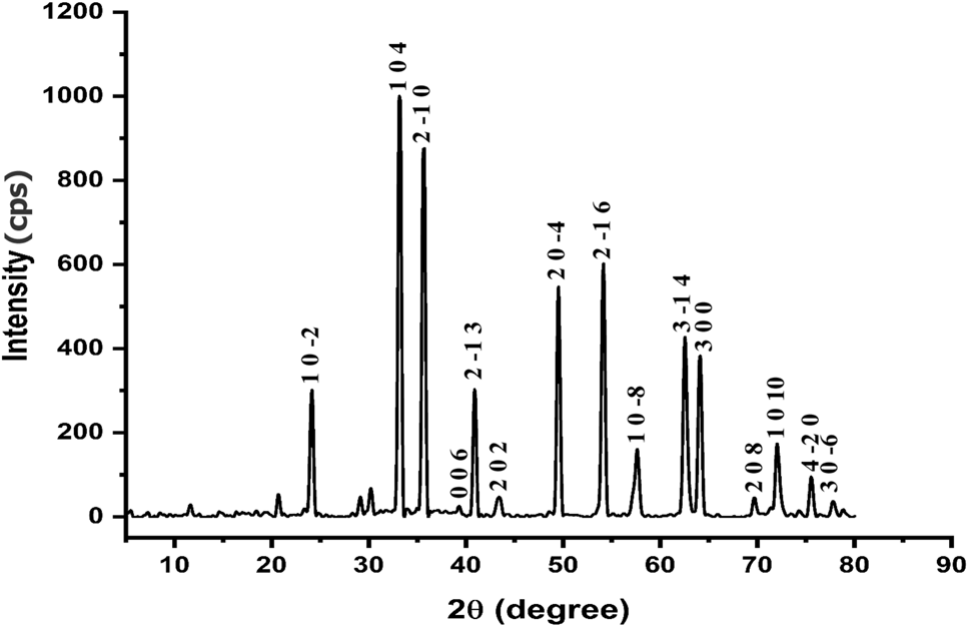
XRD diffractogram of the *α-HNPs* after Cefotaxime photocatalytic degradation.

The XRD diffractogram evidenced the highly crystalline form of *α-HNPs* after *Cfm PCD* by the Bragg's reflection peaks 2θ value and it corresponds to the lattice planes as manifested in Table 5. The diffraction of the peaks reflected the presence of Trigonal (hexagonal axes) of hematite crystal with a = 5.0346 Å; c = 13.7473 Å which is identical to the reference card ICDD No: 00-901-5964. These results are compatible with our earlier study Al-Hakkani et al.^36^ who reported the greener synthesis of *α-HNPs* highly crystalline as sharpness peaks depicted in Fig. 9 with the evidence of lattice plans. Any other presence peaks may be attributed to the *Cfm* or their related substances from the adsorption process and/or *Cfm PCD* onto the surface of *α-HNPs*^1,2^. A slight increase of the average crystallite size (**D**) 27.0 nm after the *Cfm PCD* indicates the high efficacy of the reusability of the bio-nanocatalyst *α-HNPs* for further use. Table 6 shows a comparison of the XRD parameters for the *α-HNPs* before and after *Cfm PCD*.

**Table 5:** XRD parameters of the *α-HNPs* after Cefotaxime photocatalytic degradation.

2θ˚ Reference	2θ˚_hkl_ Measured	Miller indices			D	ε	δ	α	d Reference	d Calculated
		h	k	ℓ	nm	nm	lines/nm^2^		nm	nm
24.1525	24.13781	1	0	−2	28.3	5.9 × 10^–3^	1.2 × 10^–3^	2.7 × 10^–3^	3.6818	3.6871
33.1636	33.18749	1	0	4	29.0	4.2 × 10^–3^	1.2 × 10^–3^	2.3 × 10^–3^	2.6991	2.6994
35.636	35.65609	2	−1	0	28.3	4.0 × 10^–3^	1.2 × 10^–3^	2.3 × 10^–3^	2.5173	2.5180
39.2899	39.2853	0	0	6	27.6	3.7 × 10^–3^	1.3 × 10^–3^	2.3 × 10^–3^	2.2912	2.2934
40.8679	40.90785	2	−1	3	31.9	3.1 × 10^–3^	9.8 × 10^–4^	1.9 × 10^–3^	2.2063	2.2061
43.5155	43.39244	2	0	2	20.5	4.6 × 10^–3^	2.4 × 10^–3^	2.9 × 10^–3^	2.078	2.0853
49.4705	49.52775	2	0	−4	31.0	2.7 × 10^–3^	1.0 × 10^–3^	1.8 × 10^–3^	1.8409	1.8404
54.0788	54.12368	2	−1	6	29.0	2.6 × 10^–3^	1.2 × 10^–3^	1.9 × 10^–3^	1.6944	1.6945
57.6082	57.63186	1	0	−8	20.5	3.5 × 10^–3^	2.4 × 10^–3^	2.6 × 10^–3^	1.5987	1.5994
62.4444	62.56671	3	−1	4	24.6	2.7 × 10^–3^	1.7 × 10^–3^	2.1 × 10^–3^	1.486	1.4846
64.0089	64.09551	3	0	0	28.8	2.3 × 10^–3^	1.2 × 10^–3^	1.8 × 10^–3^	1.4534	1.4528
69.6047	69.91427	2	0	8	15.9	3.8 × 10^–3^	3.9 × 10^–3^	3.2 × 10^–3^	1.3496	1.3455
71.9605	72.0675	1	0	10	24.4	2.4 × 10^–3^	1.7 × 10^–3^	2.1 × 10^–3^	1.3111	1.3105
75.4704	75.55316	4	−2	0	33.0	1.7 × 10^–3^	9.2 × 10^–4^	1.5 × 10^–3^	1.2586	1.2585
77.7497	77.81803	3	0	−6	32.5	1.7 × 10^–3^	9.5 × 10^–4^	1.5 × 10^–3^	1.2273	1.2274
Average	–	–	–	–	27.0	3.3 × 10^–3^	1.6 × 10^–3^	2.2 × 10^–3^	**–**	**–**

**Table 6:** XRD parameters comparison of the *α-HNPs* before and after Cefotaxime photocatalytic degradation.

Parameter	Before	After
Crystallite size average (D) nm	25.1	27.0
Strain average (ε)	4.0 × 10^–3^	3.3 × 10^–3^
Dislocation density average (δ) lines/nm^2^	1.6 × 10^–3^	1.6 × 10^–3^
Staking faults average (α)	2.5 × 10^–3^	2.2 × 10^–3^
Crystallinity (%)	78.8%	78.7%
Reference	Al-Hakkani et al.^36^	Current study

#### TEM analysis

**Figure 10:**
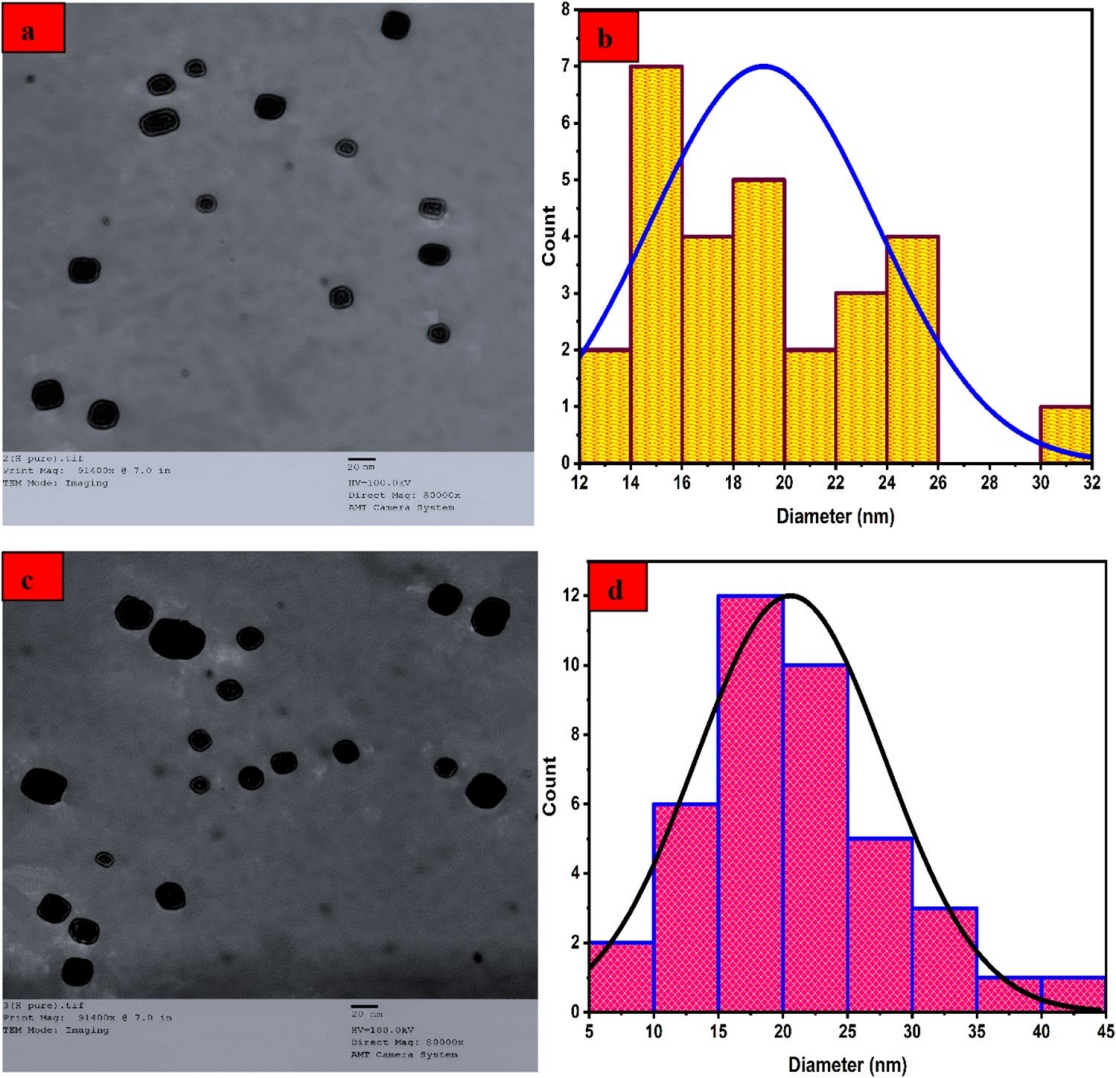
TEM analysis and particle distribution of *α-HNPs* before (**a**, **b**), after (**c**, **d**) Cefotaxime photocatalytic degradation.

The *α-HNPs* before and after *Cfm PCD* TEM analysis were being investigated as depicted in Fig. 10. Although there is an obvious very little increase in some of the particles which may be attributed to the adsorption process that was done before the degradation, but without any significant increment in particle sizes.

Monodispersed cubic shapes were being manifested without any signs of particle agglomeration. The statistical calculations of the corresponding analysis were introduced in Table 7.

**Table 7:** TEM analysis of *α-HNPs* before and after *Cfm PCD.*

Item	Before	After
Average particle sizes (nm)	19.2	20.6
Standard deviation (nm)	4.4	7.4
Minimum particle size (nm)	12.6	6.3
Maximum particle size (nm)	30.2	40.4
Median (nm)	18.8	19.8
Crystallinity index (*CI*)	1.1	0.71
Crystallinity nature	Monodispersed	

It’s clear to the man there is no change in the particle sizes of the *α-HNPs* before and after the *Cfm* photocatalytic process. This is an indication of the validity of *α-HNPs* for use to further run again without any significant change in the *α-HNPs* efficiency.

#### Morphological and chemical composition analysis (SEM and EDX)

**Figure 11:**
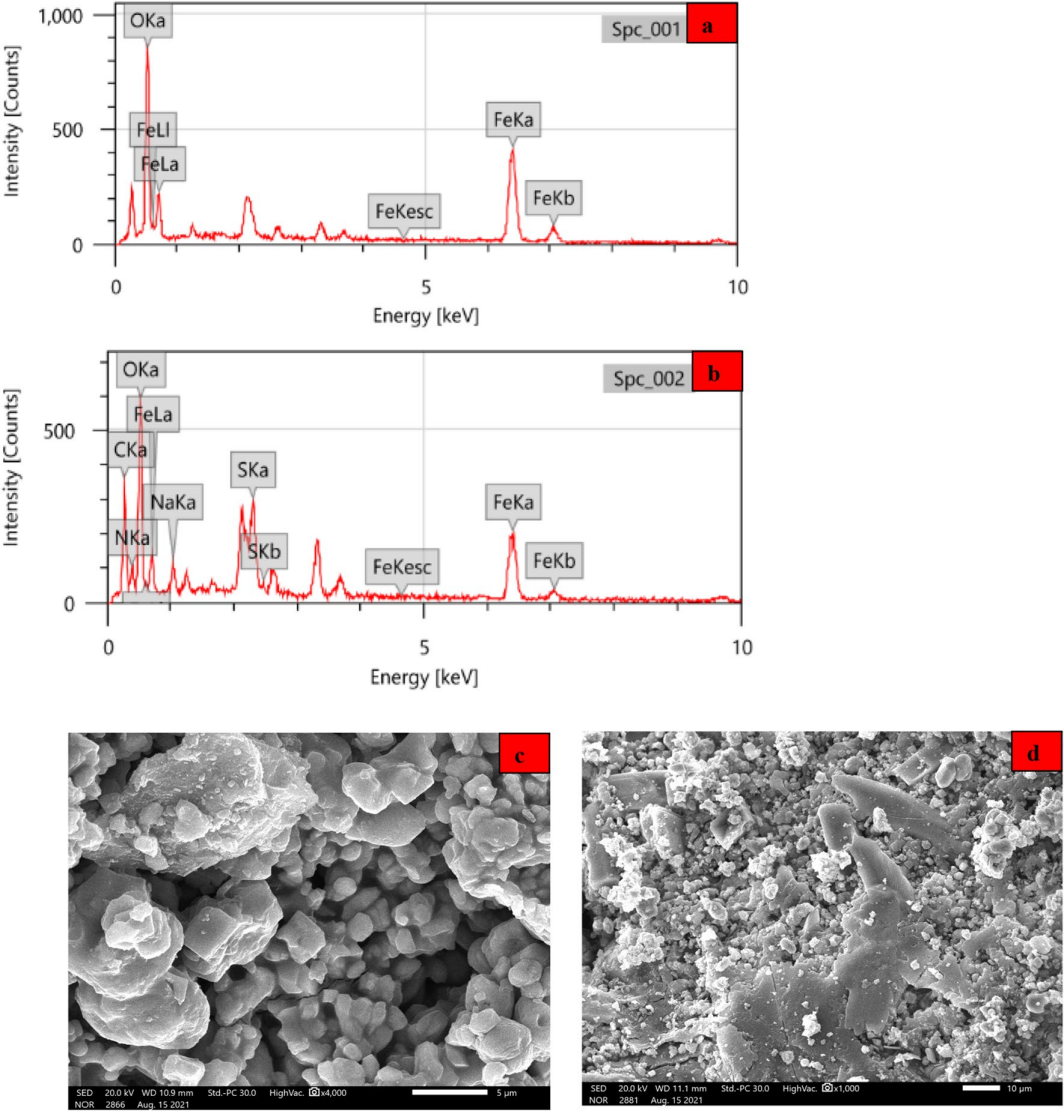
*α-HNPs* before and after Cefotaxime photocatalytic degradation EDX analysis (**a**, **b**); SEM analysis (**c**, **d**).

The surface morphology of *α-HNPs* before and after *Cfm PCD* was introduced as shown in Fig. 11. It's clear and easy for man the distinguish the surface's nature and changes that have been happened for each case of *α-HNPs.* EDX analysis showed a stronger proof to differentiate between *α-HNPs* elemental composition alone and *α-HNPs* after implementing the *Cfm PCD* process. EDX analysis in the case of *α-HNPs* without any treatment process manifested that the main element components are only oxygen and iron atoms Fig. 11A. While after the *Cfm PCD* process that occurred at the surface of the *α-HNPs* Fig. 11B we can see other elements that have appeared as carbon, nitrogen, sodium, and sulfur that are the main composition of *Cfm* and their degradation substances as it was manifested in Table 1. This result confirms the adsorption and degradation process of *Cfm* and its related substances at the *α-HNPs* surface. Also, SEM analyses for *α-HNPs* before and after the *Cfm PCD* process have been conducted showing an observable change as Fig. 11C revealed. Some of the particles are different in shapes that appeared as light-colored plates from the original dark-colored base of *α-HNPs* Fig. 11D. These white plates could be attributed to the adsorption or degradation process of *Cfm* or their related substances at the *α-HNPs* surface.

#### FT-IR analysis

**Figure 12:**
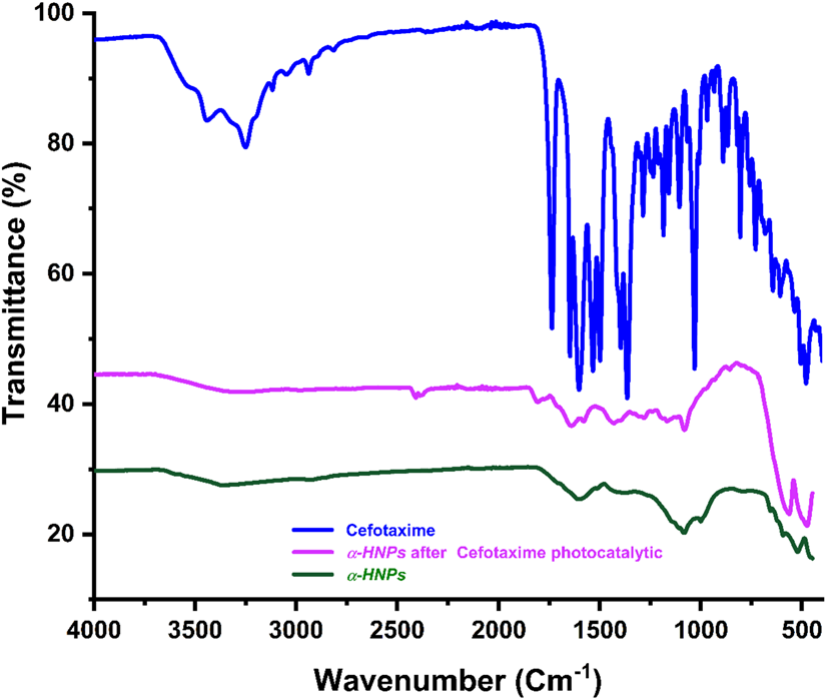
FT-IR analysis of Cefotaxime, *α-HNPs* before and after Cefotaxime photocatalytic degradation.

FT-IR analysis plays an important role to affirm the functional groups' encapsulation that adsorbed/adhered at the *α-HNPs* surface Fig. 12^2^. There are some little functional groups dedicated to *Cfm* that have clear contributions from the adsorption process at the surface of *α-HNPs* or some of the adsorbed degradant substances from *Cfm* molecules. This was exhibited especially in the fingerprint region of *Cfm* (2000–500 cm^−1^) except in the Fe–O band (522 cm^−1^)^1,36^. This investigation assures the efficacy of *α-HNPs* that could be used for multiple photocatalytic runs and this result was found to be compatible with the previously TEM findings.

#### Antibacterial activity

Results showed that DMSO had no antibacterial effect where there are no clear zones appeared. On the contrary, the aqueous extract of *E. purpurea* exhibited a moderate antibacterial activity almost was the same against both the Gram-positive and Gram-negative bacteria in the range of clear zone 12–14 mm. On the right-hand Cefotaxime manifested more activity against all tested bacterial species. The clear zones against *E. coli*, *S. albus*, and *E. faecalis* were found to be 40, 38, and 38 mm respectively where it showed a clear zone of 20 mm against *S. typhimurium*. The as-prepared *Cfm@α-HNPs* system exhibited an antibacterial activity better than *α-HNPs* alone against all tested bacterial types especially for Gram-positive bacteria more than Gram-negative bacteria.

**Figure 13:**
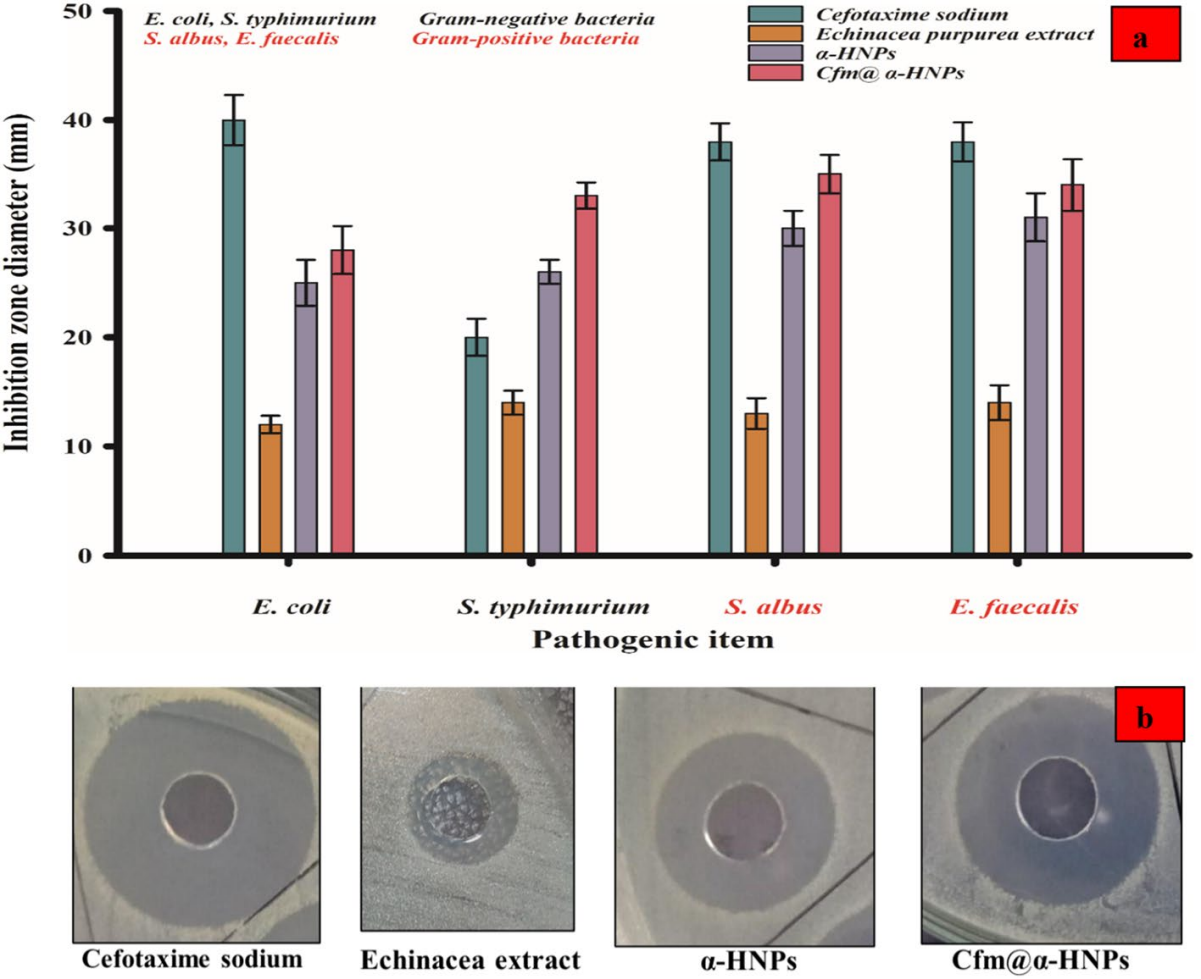
The antibacterial activity of (**a**) *E. purpurea* extract, *α-HNPs* before and after Cefotaxime photocatalytic degradation using Cefotaxime sodium as a positive control, (**b**) Representative agar diffusion showing inhibition zone diameters of the different treatments on *S. albus*.

The measurements of the inhibition clear zones were depicted in Fig. 13. The difference in the clear zones in the antibacterial activity depends on the bacterial species' ability. The results indicate that the Gram-positive bacteria are more sensitive to *α-HNPs* and *Cfm@ α-HNPs* compared to Gram-negative bacteria. This could be explained by the high ability of the as-prepared NPs to penetrate the cell wall of Gram-positive bacteria. The high activity of the *Cfm@α-HNPs* compared with *α-HNPs* alone *may* be attributed to the adsorption of some of the degradation substances after the *Cfm PCD* process that are deposited onto the *α-HNPs* surface and they have antibacterial activity. The following comparison in Table 8 showed different antibacterial activities of green biosynthesized *α-HNPs* using different plant extracts.

**Table 8:** Antibacterial activity of the biosynthesized *α-HNPs *via various parts of different kinds of plants.

Plant name/part	Application	References
*Cynometra ramiflora*	*E. coli* & *S. epidermidis*	^58^
*Carica papaya leaf*	*Klebsiella* spp., *E. Coli*, *Pseudomonas* spp. & *S. aureus*	^59^
*Sida cordifolia*	*B. subtilis*, *S. aureus*, *E. coli* & *K. pneumonia*	^64^
*Lantana camara* leaf	*P. aeruginosa*	^60^
*Terminalia bellirica*; *Moringa oleifera* fruit & *Moringa oleifera* leaves	*S. aureus*, *B. subtilis* & *P. aeruginosa*	^65^
*Skimmia Laureola* leaf	*R. solanacearum*	^66^
*Henna (Lawsonia inermis) leaf*	*S. aureus* & *S. typhimurium*	^67^
*Rheum emodi* root	*E. coli* & *S. aureus*	^61^
*Laurus nobilis L*	*L. monocytogenes*	^68^
*E. purpurea*	*E. coli*, *S. albus*, *E. faecalis* & *S. typhimurium*	Current study

#### Anti-proliferative activity

Interestingly, in addition to the antibacterial activity, the biofabricated noncompounds exhibited enhanced antiproliferative activities. IC_50_ against MCF7 cells decreased from 679.2 µg/ml when applying the plant extract to 105.31 µg/ml when *Cfm@α-HNPs* were used, pointing to marked anti-proliferative activity even compared to *α-HNPs* alone (IC_50_ = 141.6 µg/ml). A similar effect was observed using the HepG2 cells as shown in Table 9.

**Table 9:** Summary of IC_50_ of the different tested samples on the proliferation rate of MCF7 and HepG2 cell lines.

Tested product	Cell line IC_50_ (µg/ml)		References
	MCF7	HepG2	
*E. purpurea*	679.2	643.4	Al-Hakkani et al.^36^
*α-HNPs*	141.6	198.2	
*Cfm@α-HNPs*	105.31	117.33	Current study

**Figure 14:**
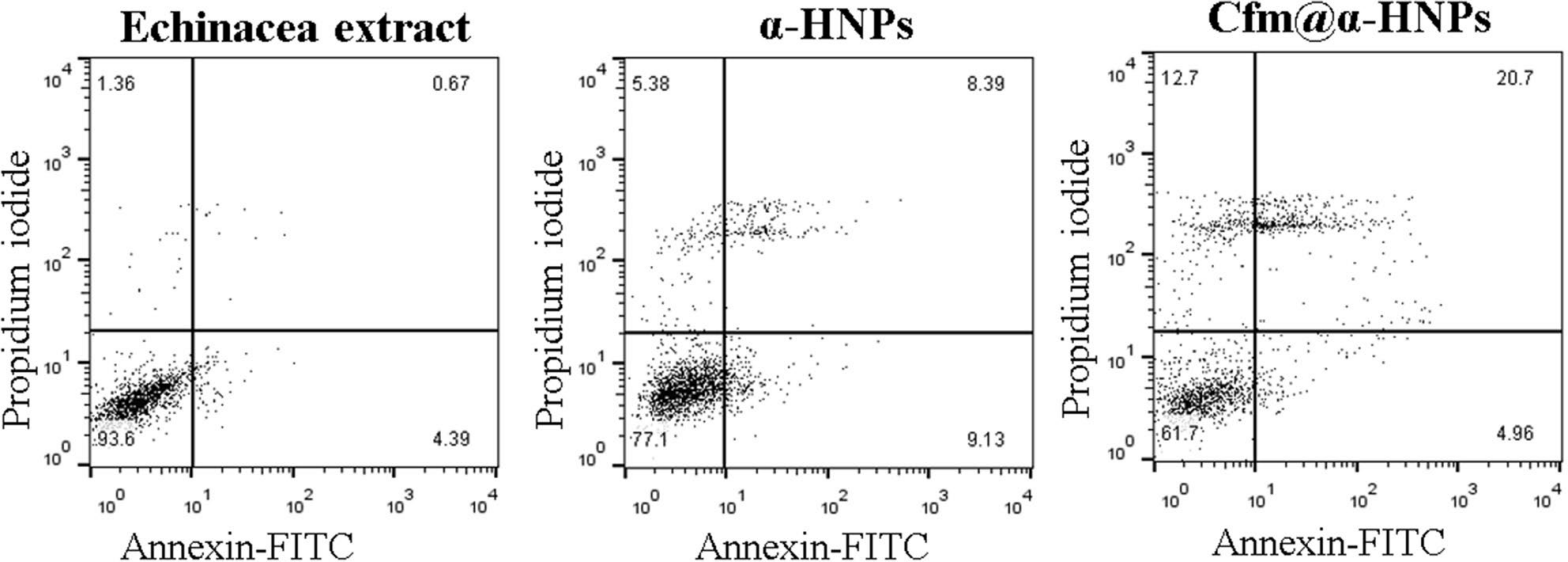
*Cfm@α-HNPs* have a marked anti-proliferative activity. MCF7 cells were treated with indicated samples and the frequency of late apoptotic cells was determined using flow cytometry.

To confirm the results of the MTT assay, cells were incubated with 100 µg/ml of plant extract, *α-HNP*, or *Cfm@α-HNPs* and the frequency of apoptotic cells was determined by flow cytometry. MCF7 cells treated with *Cfm@α-HNPs* showed the highest levels of apoptosis (20.7%) compared to cells treated with plant extract (0.67%) or *α-HNP* (8.39%) Fig. 14. Similar results were observed when HepG2 cells were tested. *Cfm@α-HNPs* and *α-HNPs* induced apoptosis in 17% and 7% of cells, respectively, while cells treated with the plant extract were almost not affected at such a low concentration of 100 µg/ml. These experiments confirmed the potential application of the biosynthesized *Cfm@α-HNPs* as a promising anti-proliferative agent.

#### Hydrogen peroxide scavenging (H_2_O_2_) assay

In the industrial process, there some properties should be available in the catalyst as the stability of photocatalytic performance that it is very vital; enhancing catalyst life and durability simplifies operations and lowers total costs. If the catalyst has a short life cycle, it will be useless and ineffective in its photocatalytic role. One of the most effective ways that could be used to estimate the photocatalytic performance of the catalysis is the scavenging assay ability.

The human immune system has a complex composition of natural enzymatic and non-enzymatic antioxidant defenses to overcome the deleterious effects of free radicals and other oxidants. Free radicals are responsible for causing a considerable majority of diseases including ulcerative colitis^69^, Alzheimer’s disease^70^, Parkinson’s disease^71^, atherosclerosis^72^, alcohol-induced liver disease^73^, cancer^74^, cardiovascular disease^75^, mild cognitive impairment^76^, aging^77^, and neural disorders^78^. Safeguarding against free radicals may be improved by a large intake of dietary antioxidants. Substantial evidence suggests that foods containing antioxidants, and probably antioxidant nutrients, in particular, may be of significant significance in the prevention of disease. Antioxidants may benefit greatly in improving the quality of life by preventing or postponing the onset of degenerative diseases.

Human beings are indirectly exposed to H_2_O_2_ through the environmental surroundings at about 0.28 mg/kg/day, mainly from leaf crops. Hydrogen peroxide may reach the human body through inhalation of vapor or mist and contact with the eyes or skin. H_2_O_2_ is quickly decomposed into oxygen and water which may create hydroxyl radicals (^•^OH) that can prompt lipid peroxidation which damages the body's DNA.

**Figure 15:**
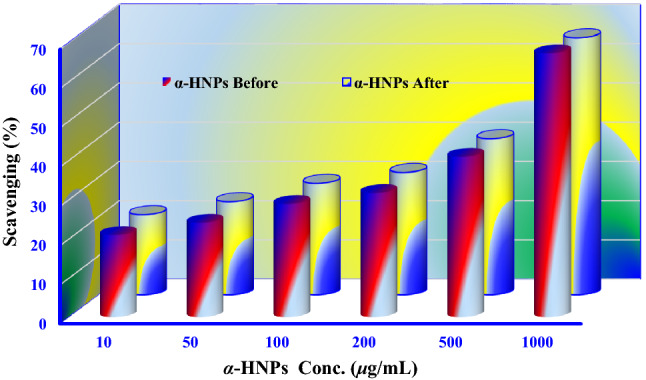
The hydrogen peroxide scavenging effect via* α-HNPs* before and after Cefotaxime photocatalytic degradation.

Figure 15 shows the hydrogen peroxide scavenging effect of the as-biofabricated *α-HNPs* before and after the photocatalytic process of *Cfm*. It was very evident from this behavior that the scavenging rate is concentration-dependent. The IC_50_ of hydrogen peroxide scavenging (%) was estimated and it was found to be 635.8 and 665.6 μg/mL of *α-HNPs* before and after the *PCD* process of *Cfm* respectively. As we can see the difference between *α-HNPs* activity before and after the photocatalytic process is less than 5%. This assures our result findings from TEM and FT-IR analysis, where the *α-HNPs* photocatalytic prosperity did not affect by the *Cfm* degradation process.

## Conclusion

In the current study, green bio-fabricated *α-HNPs* were used in efficient degradation ability to remove the *Cfm* antibiotic drug substance from the contaminated industrial wastewater. The kinetic results of the photocatalytic study manifested that was fitted to the pseudo-first-order kinetic model with R^2^ 0.992. The optimum *Cfm* photocatalytic conditions were being investigated and they were found to be at a 0.4 g/L loading dose of *α-HNPs* over 4 h of direct sunlight irradiation at solution pH 10.5. The results revealed the possibility of using the green bio-fabricated *α-HNPs* as a promising and efficient photocatalytic agent in the treatment of the aquatic contaminated environment with *Cfm*. UV–Vis confirmed that *α-HNPs* had a direct transition bandgap at 3.78 eV at a maximum absorption wavelength of 362 nm. High stability of the bio-fabricated *α-HNPs* suspension (-68.6 mV) using zeta potential was realized. XRD, TEM, SEM, EDX, and FT-IR analyses were implemented showing synergistic results for the reusability efficiency of *α-HNPs* to *Cfm* photocatalytic achievement. Promising antibacterial activity of *α-HNPs* before and after the *PCD* process of *Cfm* was investigated showing highly potent ability especially against Gram-positive pathogenic issues more than Gram-negative pathogens. Interestingly, *Cfm@α-HNPs* showed superior anti-proliferative activity as tested by MTT assay and were able to induce apoptosis in MCF7 and HepG2 cell lines as tested by Annexin-V/PI staining using the flow cytometry technique. Hydrogen peroxide scavenging activity was conducted showing a moderated effect where the IC_50_ was evaluated and it was found to be 635.8 and 665.6 μg/mL of *α-HNPs* before and after the *PCD* process of *Cfm* respectively.

## Supplementary Information

Supplementary Information.

## Data Availability

The data used to support the findings of this study are included in the article.
