# Supplementary material for: Cefotaxime removal enhancement via bio-nanophotocatalyst α-Fe2O3 using photocatalytic degradation technique and its echo-biomedical applications
Source: Sci Rep. 2022 Jul 13;12:11881. doi: 10.1038/s41598-022-14922-3 (PMC9279508; doi:10.1038/s41598-022-14922-3)
Supplement: Supplementary file 1 — Supplementary Information. [file 41598_2022_14922_MOESM1_ESM.docx]

1. ***XRD parameters calculation***

***D = 0.9λ / β cos(θ) (1)***

Where ***D*** is the average crystallite size, ***λ*** is the wavelength for the used XRD radiation source which equals = 0.1541838 nm, ***β*** is the corrected full widths at half maxima of the measured peaks, and ***θ*** is the Bragg’s angle diffraction.

***ℇ = β / 4 tan(θ) (2)***

***d = λ / 2 sin(θ) (3)***

***δ = 1/D^2^ in lines / nm^2^ (4)***

***α = [(2π^2^) / (45(3 tan(θ))^0.5^)] β (5)***

***Crystallinity (%) = C_A_ / T_A_ x 100 (%) (6)***

***C_A_*** is summation of the crystalline peak areas in the diffractogram.

***T_A_*** is summation of the crystalline and amorphous peak areas in the diffractogram.

***CI = I_1_/I_2_ (7)***

***I_1_*** represents the particle size obtained from TEM analysis.

***I_2_*** represents the crystallite size for the principle peak of “Miller indices at hkl" obtained from XRD analysis using the Scherrer equation.

1. ***Photocatalytic degradation approach***

***Degradation efficiency (%) = (C_0_-C_t_)/ C_0_ x 100 (8)***

Where C_0_ is the initial concentration and C_t_ is the founded concentration at time t of *Cfm*.

***ln (C_0_/ C_t_) = K_1_t (9)***

***(1/ C_t_) - (1/ C_0_ )= K_2_t (10)***

Where C_0_ is the initial concentration (mg L^-1^), C_t_ is the founded concentration (mg L^-1^) of *Cfm*, t (hr) is the reaction time, K_1_ (hr^-1^), and K_2_ (L mg^-1^ hr^-1^) represents the ﬁrst order and second-order reaction rate constant.

1. ***Hydrogen peroxide scavenging (H_2_O_2_) assay***

***Scavenging (%) = (A_S_ -A_T_)/ A_T_ x 100 (11)***

Where A_S_ represents the absorbance of the H_2_O_2_ standard and A_T_ represents the absorbance of the *α-HNPs* test.

1. ***UV-Vis. analysis***

***α = 2.303*A/ d (12)***

Where; *α* is the absorption coefficient, d is the thickness of the sample, and A is the absorbance.

***(α hυ)^n^ = C (hυ- E_g_) (13)***

Where; *α* is the absorption coefficient, hυ is the photon energy. n is assumed values of 2 and 0.5 for direct and indirect transitions respectively, C is energy independent constant and E_g_ is the energy band gap.
